# ICU-Mortality in Old and Very Old Patients Suffering From Sepsis and Septic Shock

**DOI:** 10.3389/fmed.2021.697884

**Published:** 2021-07-09

**Authors:** Raphael Romano Bruno, Bernhard Wernly, Behrooz Mamandipoor, Richard Rezar, Stephan Binnebössel, Philipp Heinrich Baldia, Georg Wolff, Malte Kelm, Bertrand Guidet, Dylan W. De Lange, Daniel Dankl, Andreas Koköfer, Thomas Danninger, Wojciech Szczeklik, Sviri Sigal, Peter Vernon van Heerden, Michael Beil, Jesper Fjølner, Susannah Leaver, Hans Flaatten, Venet Osmani, Christian Jung

**Affiliations:** ^1^Division of Cardiology, Pulmonology and Vascular Medicine, Medical Faculty, University Hospital Düsseldorf, Heinrich-Heine-University Düsseldorf, Düsseldorf, Germany; ^2^Department of Anesthesiology, Perioperative Medicine and Intensive Care Medicine, Paracelsus Medical University of Salzburg, Salzburg, Austria; ^3^Center for Public Health and Healthcare Research, Paracelsus Medical University of Salzburg, Salzburg, Austria; ^4^Department of Cardiology, Paracelsus Medical University of Salzburg, Salzburg, Austria; ^5^Fondazione Bruno Kessler Research Institute, Trento, Italy; ^6^Hôpitaux de Paris, Hôpital Saint-Antoine, Service de Réanimation Médicale, Paris, France; ^7^Sorbonne Universités, UPMC Univ Paris 06, UMR_S 1136, Institut Pierre Louis d'Epidémiologie et de Santé Publique, Paris, France; ^8^INSERM, UMR_S 1136, Institut Pierre Louis d'Epidémiologie et de Santé Publique, Paris, France; ^9^Department of Intensive Care Medicine, University Medical Center, University Utrecht, Utrecht, Netherlands; ^10^Intensive Care and Perioperative Medicine Division, Jagiellonian University Medical College, Kraków, Poland; ^11^Medical Intensive Care Unit, Hadassah University Hospital, Jerusalem, Israel; ^12^General Intensive Care Unit, Hadassah University Hospital, Jerusalem, Israel; ^13^Department of Intensive Care, Aarhus University Hospital, Aarhus, Denmark; ^14^Research Lead Critical Care Directorate St George's Hospital, London, United Kingdom; ^15^Department of Intensive Care, Anesthesia and Surgical Services, Haukeland University Hospital Bergen, Bergen, Norway

**Keywords:** sepsis, intensive care, critically ill, obesity, old, very old, octogenarian, geriatric

## Abstract

**Purpose:** Old (>64 years) and very old (>79 years) intensive care patients with sepsis have a high mortality. In the very old, the value of critical care has been questioned. We aimed to compare the mortality, rates of organ support, and the length of stay in old vs. very old patients with sepsis and septic shock in intensive care.

**Methods:** This analysis included 9,385 patients, from the multi-center eICU Collaborative Research Database, with sepsis; 6184 were old (aged 65–79 years), and 3,201 were very old patients (aged 80 years and older). A multi-level logistic regression analysis was used to fit three sequential regression models for the binary primary outcome of ICU mortality. A sensitivity analysis in septic shock patients (*n* = 1054) was also conducted.

**Results:** In the very old patients, the median length of stay was shorter (50 ± 67 vs. 56 ± 72 h; *p* < 0.001), and the rate of a prolonged ICU stay was lower (>168 h; 9 vs. 12%; *p* < 0.001) than the old patients. The mortality from sepsis was higher in very old patients (13 vs. 11%; *p* = 0.005), and after multi-variable adjustment being very old was associated with higher odds for ICU mortality (aOR 1.32, 95% CI 1.09–1.59; *p* = 0.004). In patients with septic shock, mortality was also higher in the very old patients (38 vs. 36%; aOR 1.50, 95% CI 1.10–2.06; *p* = 0.01).

**Conclusion:** Very old ICU-patients suffer from a slightly higher ICU mortality compared with old ICU-patients. However, despite the statistically significant differences in mortality, the clinical relevance of such minor differences seems to be negligible.

## Introduction

Sepsis is common and is associated with a high morbidity and mortality (1–5). During the last two decades, improvements in intensive care therapy have lowered the mortality from sepsis. However, critically ill old (>64 years), and very old (>79 years) patients are more at risk, with older patients developing sepsis more frequently and with greater severity (6, 7).

This risk is of great importance for intensive care medicine as old and very old patients are among the fastest-growing subgroups of all patients admitted to the intensive care unit (ICU) (8). Furthermore, in the European Union approximately 24 million people will be age 85 years or older by 2040 (9). Over the last decade this demographic development has already affected the admission profile to ICU (10). From a public health standpoint, aside from the ethical issues, given the significant cost associated with intensive care, unwanted and medically inappropriate intensive care admissions could result in a misallocation of valuable resources (11, 12). On one hand intensive care medicine is associated with high costs, additional suffering due to invasive procedures and a loss of dignity but on the other hand, intensive care triage based on chronological age alone has been heavily criticized (13–15).

These issues result in ongoing conflict for the intensive care physician: There are increasingly older and frailer patients, more and more intensive care treatments available, but at the same time a decreasing overall capacity due to economic constraints and more recently the covid pandemic.

However, in reality, in situations, such as a pandemic, the chronological age often serves as a key factor used to estimate the predicted outcome for a critically ill patient and thus whether they are admitted to ICU (16). Despite this, we do not know whether being “very old” compared with being “old” is a risk factor for a worse outcome.

Therefore, this study aimed to investigate and compare the mortality of old and very old patients with sepsis. Furthermore, we compared the rates of organ support and the length of stay between these two groups. We conducted this analysis using the multi-center eICU Collaborative Research Database (17).

## Methods

### Database

The eICU-Database was originally drawn from the eICU telehealth system. This system complemented on-site ICU teams with remote support. This multi-center ICU database, comprised over 200,000 admissions to 335 ICUs from 208 hospitals across the USA in 2014 and 2015 (17). Patient demographics available in the eICU database included age, sex, ethnicity, vital signs, diagnoses, laboratory measurements, clinical history, problem lists, APACHE IVa score, and treatment.

### Study Subjects

Septic patients in this study were identified *via* billing codes using the method established by Angus et al. (18). Septic shock was defined according to the Sepsis 3 definition (5). In total, 9,385 patients with sepsis over the age of 64 were included in this analysis from the eICU Collaborative Research Database.

### Data Collection

We extracted baseline characteristics and management strategies (defined as use of vasopressors and mechanical ventilation) on day one. The (pre-defined) site of primary infection and the ethical background were extracted. The database was released under the Health Insurance Portability and Accountability Act (HIPAA) safe harbor provision. The re-identification risk was certified as meeting safe harbor standards by Privacert (Cambridge, MA) (HIPAA Certification no. 1031219-2).

### Statistical Analysis

Continuous data are expressed as median ± interquartile range. We assessed differences between independent groups using Kruskal-Wallis equality-of-populations rank test. We expressed categorical data as numbers (percentage) and used the Chi-square test to calculate univariate differences between groups.

The primary exposure was the age dichotomised in two age strata: old patients, i.e., patients aged 65–79 years (*n* = 6,184), and very old patients, i.e., patients aged 80 years and older (*n* = 3,201). The primary outcome of our analysis was ICU mortality. As secondary outcomes we evaluated the frequency of mechanical ventilation and vasopressor use. We used multi-level logistic regression to fit three sequential regression models for the binary primary outcome to assess the impact of the age category on ICU-mortality. First, a baseline model with the age category as a fixed effect and ICU as a random effect (model-1) was fitted. Second, to model-1, patient characteristics (BMI, SOFA score, gender, infection source, ethnicity) (model-2) were added as independent variables to the model. Third, to model-2, management strategies (mechanical ventilation and vasopressor use; model-3) were added to the model. Model-1 and model-2 were used to evaluate the primary and secondary outcomes, whereas model-3 was only used to assess the primary outcome. We chose the independent variables based on previous reports and our own clinical experience. We calculated adjusted odds ratios (aOR) with respective 95% confidence intervals (95%CI).

We conducted all analyses in the total cohort of 9,385 patients diagnosed with sepsis and a sub-group of patients with septic shock (*n* = 1,054).

Additionally, we performed stratified sensitivity analyses, stratifying patients receiving above and below 30 ml/kg/h of fluid, creatinine above and below 2.0 mg/dl (arbitrary cut-off), lactate above and below 2.0 mmol/L (arbitrary cut-off), SOFA > 1 (Sepsis-3 criteria) and SOFA > 6 (median SOFA score), with and without mechanical ventilation, with and without the vasopressor use, and patients with a primary pulmonary focus vs. non-pulmonary (all other foci), and Caucasian patients (being the most frequent ethnic group) vs. non-Caucasian patients. We performed the stratified sensitivity analyses using model-1. Length of stay was divided into <72, 72–168, and >168 h.

All tests were two-sided, and a *p*-value of <0.05 was considered statistically significant. We used Stata/IC 16.1 (StataCorp. 2019. Stata Statistical Software: Release 16. College Station, TX, USA: StataCorp LLC) for all the statistical analyses.

## Results

### Total Cohort of Septic Patients

In the total cohort of 9,385 patients, 6,184 were old patients (aged 65–79 years), and 3,201 were very old patients (aged 80 years and older). The baseline characteristics of old vs. very old patients are shown in Table 1A. Very old patients had a lower BMI (25 ± 8 vs. 28 ± 10; *p* < 0.001), and a higher serum creatinine concentration (1.4 ± 1.5 vs. 1.5 ± 1.3; *p* = 0.005). The baseline serum lactate concentration (2.0 ± 1.9 vs. 1.8 ± 1.8; *p* = 0.02), as well as the rate of patients with an increased serum lactate (>2.0 mmol/L; 47 vs. 44%; *p* = 0.02), were higher in the very old. The primary source of infection differed between groups. Very old patients suffered from urinary tract infections significantly more frequently [1,490 (24%) vs. 887 (28%), *p* < 0.001], but significantly less from skin infections [477 (8%) vs. 160 (5%), *p* < 0.001]. Ethnic background also differed. For example, the proportion of African Americans was higher in the old patients [540 (9%) vs. 204 (6%), *p* < 0.001], while the proportion of Hispanics was greater in the very old patients [193 (3%) vs. 149 (5%), *p* < 0.001]. However, these differences were not detected when the subgroup of patients with septic shock was analyzed.

**Table 1:** Baseline characteristics in the total cohort **(A)** and the subgroup of patients with septic shock **(B)** stratified for age categories into old and very old septic patients.

	**A Total cohort**			**B Septic shock**		
	**Old (>64 years)**	**Very old (>79 years)**	***p*-value**	**Old (>64 years)**	**Very old (>79 years)**	***p*-value**
	***n* = 6,184**	***n* = 3,201**		***n* = 730**	***n* = 324**	
Age (years); median (IQR)	72 (8)	84 (4)	<0.001	72 (8)	84 (4)	<0.001
male; *n* (%)	3,221 (52)	1,638 (51)	0.40	384 (53)	177 (55)	0.54
BMI; median (IQR)	28 (10)	25 (8)	0.001	27 (10)	25 (8)	<0.001
BMI <18.5	289 (14)	217 (15)	0.48	29 (12)	24 (15)	0.31
BMI > 30	2,243 (56)	740 (40)	<0.001	255 (54)	75 (36)	<0.001
SOFA score; median (IQR)	6 (5)	6 (5)	0.66	10 (5)	9 (4)	0.007
Creatinine (mg/dl); median (IQR)	1.4 (1.5)	1.5 (1.3)	0.005	2.1 (1.6)	1.9 (1.5)	0.13
Creatinine >2.0mg/dl	1,809 (31)	911 (31)	0.82	369 (52)	142 (46)	0.052
Lactate (mmol/L); median (IQR)	1.8 (1.8)	2.0 (1.9)	0.02	3.8 (3.5)	3.7 (3.1)	0.18
**Infection focus**						
UTI; *n* (%)	1,490 (24)	887 (28)	<0.001	158 (21)	90 (28)	0.03
Pulmonary; *n* (%)	2,407 (39)	1,264 (40)	0.60	261 (36)	102 (32)	0.18
GI; *n* (%)	730 (12)	382 (12)	0.85	142 (20)	50 (15)	0.12
Cutaneous; *n* (%)	477 (8)	160 (5)	<0.001	32 (4)	16 (5)	0.69
Unknown; *n* (%)	704 (11)	328 (10)	0.10	95 (13)	44 (14)	0.80
Gynaecologic; *n* (%)	7 (<1)	0 (0)	0.06	1 (<1)	0 (0)	0.51
Other; *n* (%)	369 (6)	180 (6)	0.50	41 (6)	22 (7)	0.46

*SOFA, Sepsis-related organ failure assessment; BMI, body mass index; UTI, urinary tract infection; GI, gastrointestinal*.

**Figure 1:**
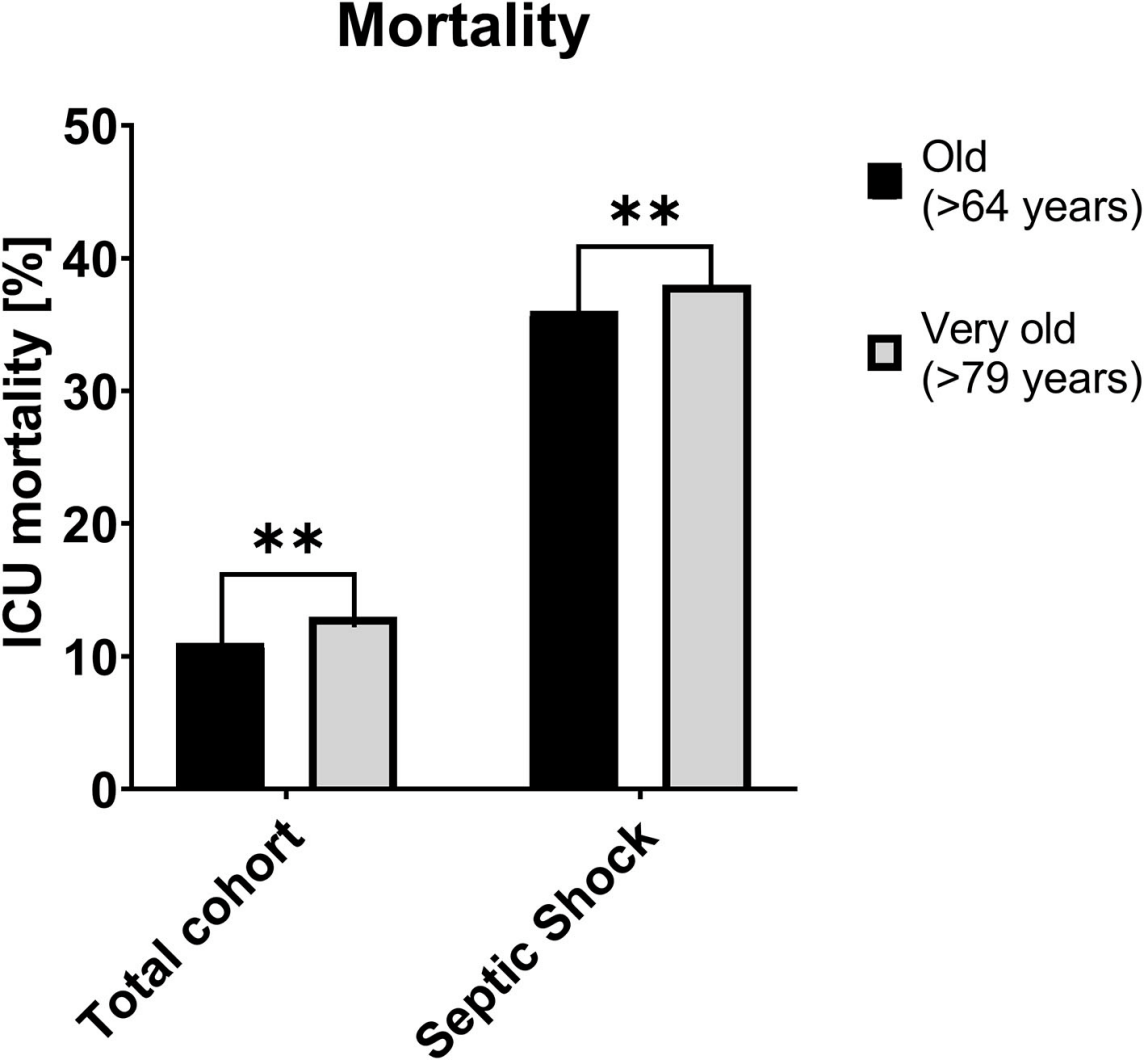
ICU-Mortality of the total cohort **(left)** and the subgroup for patients with septic shock **(right)**, [%] **p < 0.01. ICU, Intensive care unit.

The median length of stay was shorter (50 ± 67 h vs. 56 ± 72; <0.001), and the rate of short-term stay (<72 h; 65 vs. 62%; *p* < 0.001) was higher in the very old (Figure 1 left). Also, the rate of a prolonged stay was lower in the very old (>168 h; 9 vs. 12%; *p* < 0.001). We could not detect significant differences in the relative amounts of fluid administered (Table 2).

**Table 2:** Length of stay and amount of fluid in in the total cohort **(A)** and the subgroup of patients with septic shock **(B)** stratified for age categories into old and very old septic patients.

	**A Total cohort**			**B Septic shock**		
	**Old (>64 years)**	**Very old (>79 years)**	***p*-value**	**Old (>64 years)**	**Very old (>79 years)**	***p*-value**
	***n* = 6,184**	***n* = 3,201**		***n* = 730**	***n* = 324**	
Length of stay (h); median (IQR)	56 (72)	50 (67)	<0.001	71 (110)	64 (84)	0.01
<72 h; *n* (%)	3,805 (62)	2,093 (65)	<0.001	374 (51)	181 (56)	0.17
72–168 h; *n* (%)	1,612 (26)	814 (25)	0.50	193 (26)	105 (32)	0.05
>168 h; *n* (%)	767 (12)	294 (9)	<0.001	163 (22)	38 (12)	<0.001
**Fluid management in first 24 h**						
Total amount of fluid (ml); median (IQR)	2,570 (2,890)	2,430 (2,608)	0.03	3,270 (3,815)	3,405 (3,258)	0.99
Amount of fluid per kg bodyweight; median (IQR)	32 (38)	33 (39)	0.10	40 (51)	47 (55)	0.15
Amount of fluid per kg BW > 30ml/kg/h; *n* (%)	1,581 (52)	865 (54)	0.17	235 (61)	110 (69)	0.10

*SOFA, Sepsis-related organ failure assessment; BMI, body mass index; UTI, urinary tract infection; GI, gastrointestinal*.

The rate of vasopressor use was comparable (33 vs. 34%; Table 3) between old and very old patients, but the rate of mechanical ventilation was lower in very old patients (18 vs. 23%), and this association remained after adjustment for both the random effect in model-1 as well as patient-specific characteristics in model-2.

**Table 3:** Associations of old vs. very old septic patients with mortality and management strategies in three multi-level logistic regression models.

**Total cohort**					
	**Crude events**				
	**Old**	**Very old**	**Model 1**	**Model 2**	**Model 3**
	***n*** **(%)**	***n*** **(%)**	**aOR (95% CI**, ***p*****-value)**	**aOR (95% CI**, ***p*****-value)**	**aOR (95% CI**, ***p*****-value)**
ICU mortality	692 (11)	412 (13)	1.21 (1.06–1.38; 0.005)	1.28 (1.06–1.54; 0.01)	1.32 (1.09–1.59; 0.004)
Management	–	–	–	–	–
Mechanical ventilation	1,418 (23)	562 (18)	0.72 (0.64–0.81; <0.001)	0.72 (0.61–0.85; <0.001)	–
Vasopressor use	2,075 (34)	1,045 (33)	0.99 (0.90–1.10; 0.91)	0.99 (0.86–1.14; 0.86)	–

*Model 1, ICU cluster as random effect; Model 2, Model 1 plus patient level (SOFA, BMI, sex, ethnics, infection focus, lactate concentration); Model 3, Model 2 plus management strategies (mechanical ventilation, vasopressor use); SOFA, Sepsis-related organ failure assessment; BMI, body mass index*.

**Figure 2:**
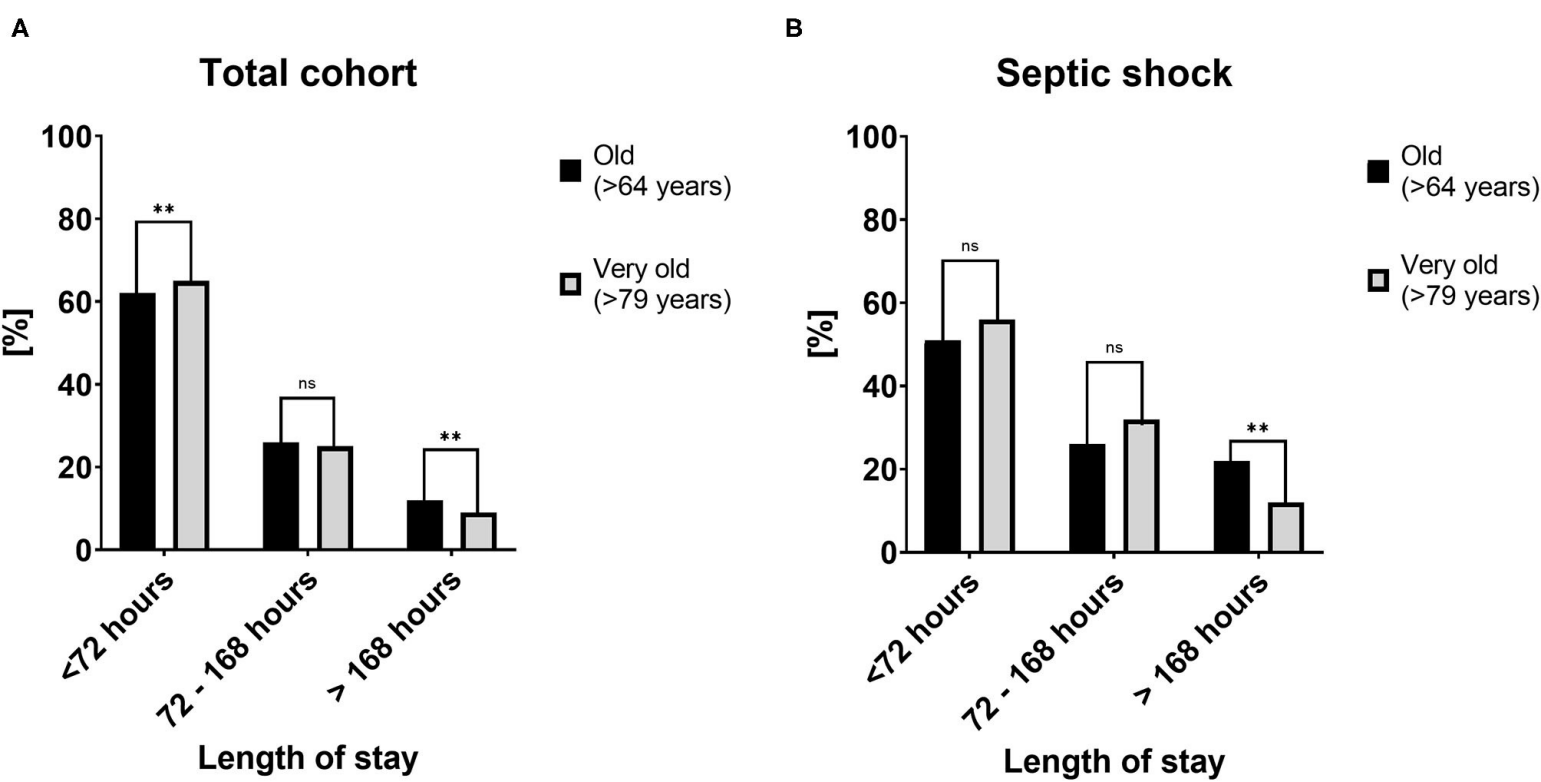
Length of stay in the total cohort **(A)** and the subgroup of patients with septic shock **(B)** stratified for length of stay categories into <72, 72–168, and >168 h. [%] **p < 0.01.

ICU-mortality was higher in the very old (13 vs. 11%, Figure 2) and being very old was associated with a higher odd for ICU mortality in model-1 (aOR 1.21 95% CI 1.06–1.38; *p* = 0.005); model-2 (aOR 1.28 95% CI 1.06–1.54; *p* = 0.01) and model-3 (aOR 1.32 95% CI 1.09–1.59; *p* = 0.004).

**Figure 3:**
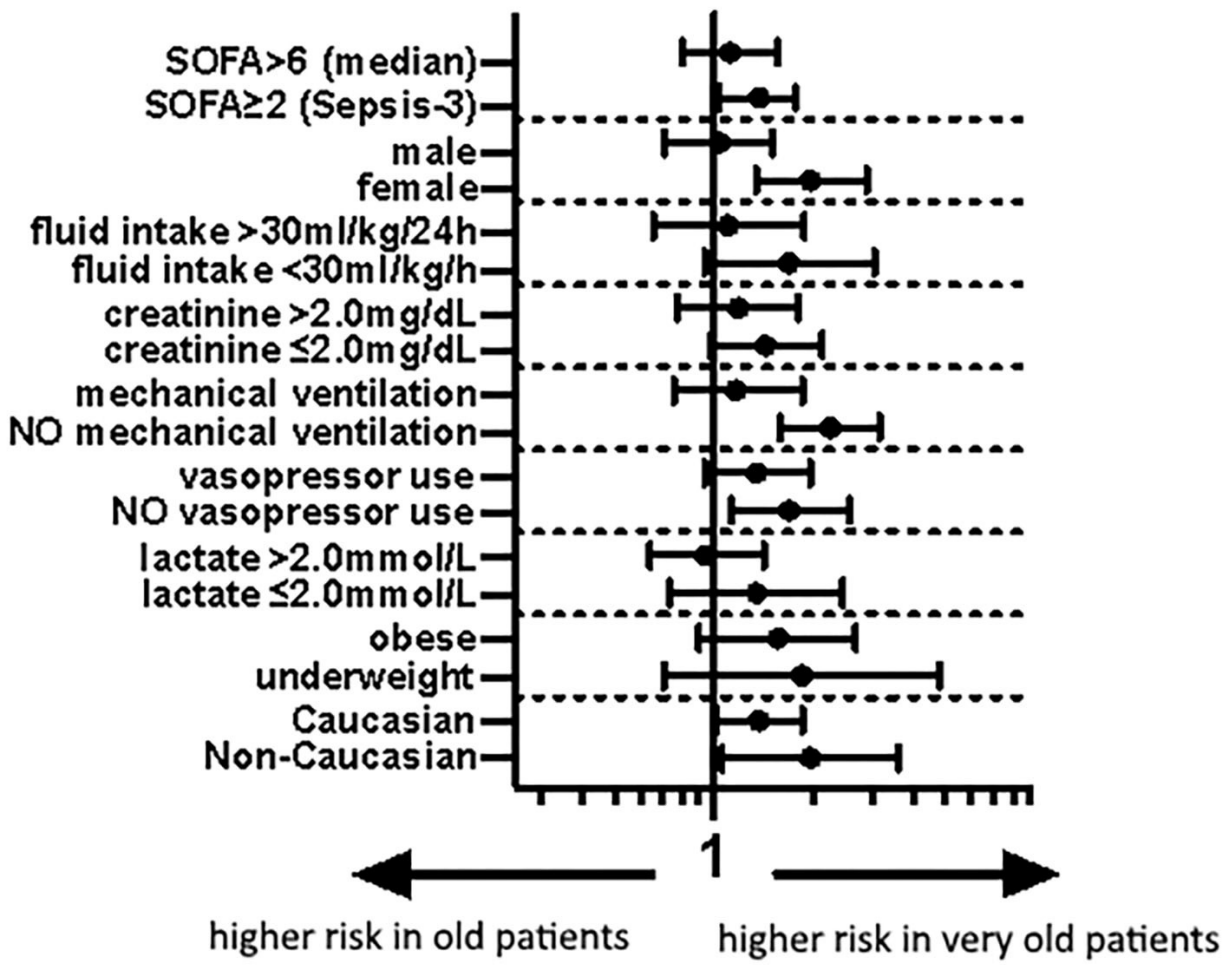
Forest plot of aOR of old vs. very old septic patients for different subgroups according to model-1 (aOR 95% CI). SOFA, Sepsis-related organ failure assessment.

In the sensitivity analyses, being very old was associated with a higher odds of ICU mortality in female patients (aOR 1.40 95%CI 1.16-1.70), non-ventilated patients (aOR 1.50 95% CI 1.26–1.78) and patients without vasopressor use (aOR 1.30 95% CI 1.06–1.60; Figure 3).

### Sub-cohort of Patients With Septic Shock

In the sub-group of patients with septic shock, according to Sepsis-3, 730 patients were old and 324 very old. The very old patients evidenced lower median BMI values (27 ± 10 vs. 25 ± 8; *p* < 0.001) and lower SOFA scores (10 ± 5 vs. 9 ± 4; *p* = 0.007). The median baseline creatinine (1.9 ± 1.5 vs. 2.1 ± 1.6; *p* = 0.13), and lactate (3.7 ± 3.1 vs. 3.8 ± 3.5; *p* = 0.18) concentrations were similar (Table 1B).

The length of stay was lower in the very old patients (64 ± 84 vs. 71 ± 110 h; *p* = 0.01), and the rate of short-term stay (<72 h; 56 vs. 51%; *p* = 0.17) was higher, and the rate of long-term stay (>168 h; 12 vs. 22%; *p* < 0.001) was lower. There was no difference in fluid management (Table 2B). The mechanical ventilation rates were significantly lower in the very old patients (42 vs. 55%; *p* < 0.001).

**Figure 4:**
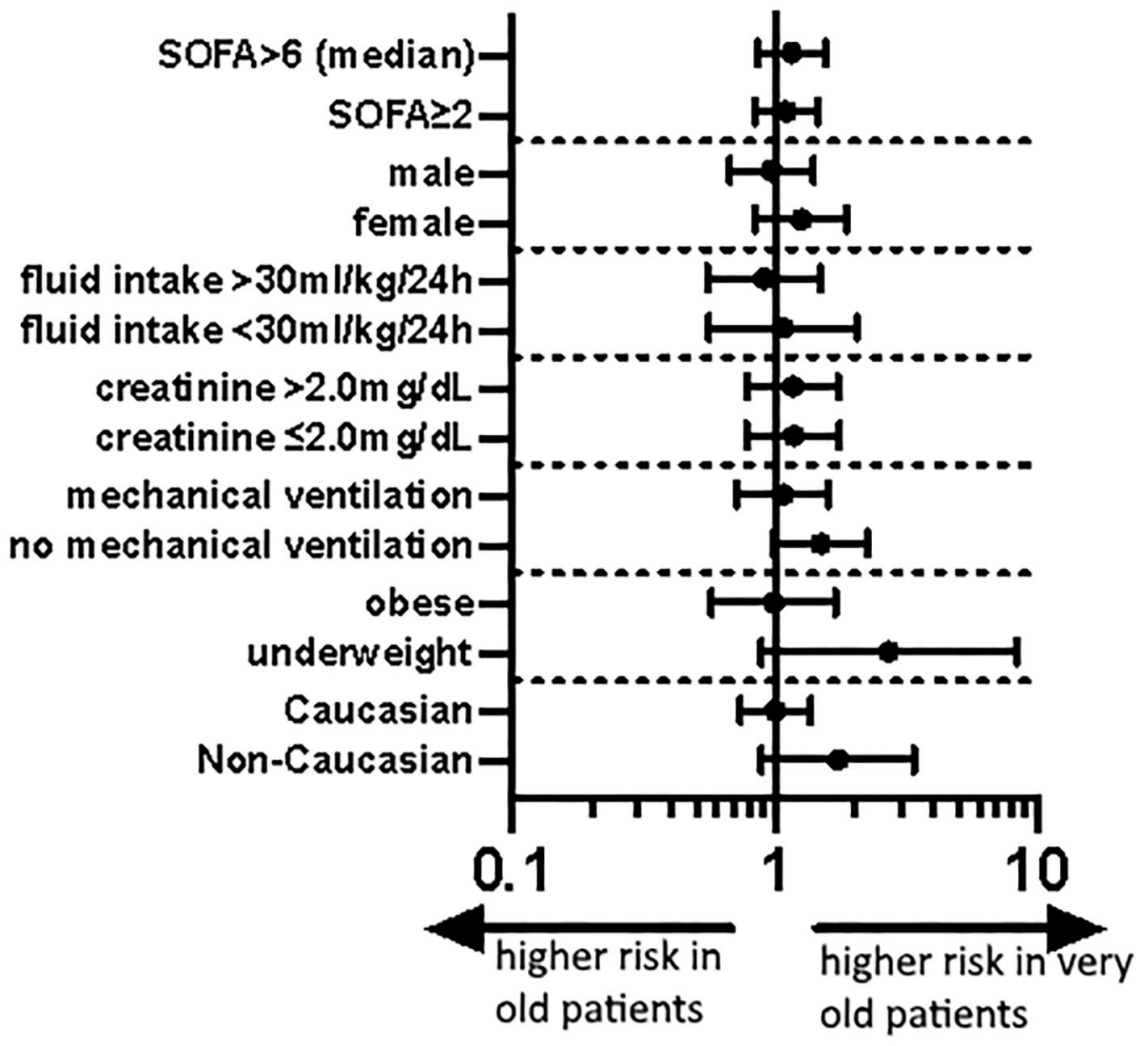
Forest plot of aOR of old vs. very old septic shock patients for different subgroups according to model-1 (aOR 95% CI). SOFA, Sepsis-related organ failure assessment.

Mortality was higher in the very old patients (38 vs. 36%) and being very old remained associated with a higher odds of ICU mortality after adjustments in model-1 (aOR 1.10 95% CI 0.84–1.45; *p* = 0.49), model-2 (aOR 1.46 95% CI 1.07–1.99; *p* = 0.02) and model-3 (aOR 1.50 95% CI 1.10–2.06; *p* = 0.01, Table 4). The stratified sensitivity analyses are shown in Figure 4.

**Table 4:** Association of old vs. very old septic shock patients with mortality and management strategies in three multi-level logistic regression models.

**Septic shock patients**					
	**Crude events**				
	**Old**	**Very old**	**Model 1**	**Model 2**	**Model 3**
	***n*** **(%)**	***n*** **(%)**	**aOR (95% CI**, ***p*****-value)**	**aOR (95%CI**, ***p*****-value)**	**aOR (95% CI**, ***p*****-value)**
ICU mortality	260 (36)	122 (38)	1.10 (0.84–1.45; 0.49)	1.46 (1.07–1.99; 0.02)	1.50 (1.10–2.06; 0.01)
Management	–	–	–	–	–
Mechanical ventilation	404 (55)	136 (42)	0.60 (0.46–0.79; <0.001)	0.68 (0.49–0.93; 0.02)	–
Vasopressor use	730 (100)	324 (100)	–	–	–

*Model 1, ICU cluster as random effect; Model 2, Model 1 plus patient level (SOFA, BMI, sex, ethnics, infection focus, lactate concentration); Model 3, Model 2 plus management strategies (mechanical ventilation, vasopressor use); SOFA, Sepsis-related organ failure assessment; BMI, body mass index*.

## Discussion

In this retrospective multi-center study of old (65–79 years) vs. very old (aged 80 and older) critically ill patients with sepsis admitted to ICU, we found a slightly higher ICU mortality in the very old. Recently, in Europe during the Covid-19 pandemic, as part of triage, numerous ICUs set an age cut-off for ICU admission (16). This study shows that although there is a difference in mortality, it is very small–and probably clinically negligible.

Sepsis is a common acute illness associated with a high mortality–some authors estimate that sepsis is the third leading cause of death in the Western world (1–4). The key elements to successful treatment of sepsis are: intelligent fluid management, early antibiotics, source control administration of catecholamines, and early diagnosis of the disease using scientifically based definitions (19, 20). For our analysis, we used the Angus criteria to detect septic patients from a large electronic database of critically ill patients (18). This yielded a large cohort of patients with a relatively low absolute mortality compared to other cohorts evaluating old septic patients—for example, Ibarz et al. (21) recently reported a 43% 30 day-mortality in very old septic patients. The use of the Angus criteria might therefore constitute a limitation to our analysis. However, the Angus criteria are well established for explorative analyses in large databases, and furthermore the results were consistent both in the sensitivity analysis applying Sepsis-3 criteria for sepsis (i.e., SOFA ≥ 2 points), as well as Sepsis-3 criteria for septic shock. We therefore think that our finding of a relatively modest effect of being old or very old on ICU mortality in septic patients exists regardless of the applied criteria for sepsis.

Our results are at odds with previous analyses, which demonstrated an effect of age on mortality in septic patients (22, 23). However, Martin-Loeches et al. (24) also found that age was not an independent risk factor in old patients (≥65 years).

In our analysis, very old patients were intubated and ventilated significantly less frequently than “old only” patients. This may indicate that in these very old patients–even after adjusting for numerous confounders–the decision to limit therapy was made more frequently on the basis of age. In fact, we found a significantly lower rate of mechanical ventilation in the very old, although the median SOFA and the numbers with a pulmonary focus for sepsis were similar in both the old and the very old. These findings indicate a less intensive approach to treatment in the very old and support previous analysis. Boumendil et al. (25), reported less intensive treatment even after adjustment for organ failure and the severity of sickness in old patients. We can only speculate about the reasons for this–on the one hand, pre-defined treatment goals might prevent more aggressive treatment in the very old; on the other hand, physicians might unconsciously be more reluctant to use organ replacement therapies in very old patients. In addition, many older patients often choose to avoid unnecessary prolongation of life by organ support and intensive care. Based on these considerations, the value of intensive care treatment has been questioned in the very old (12, 26). Heyland et al. (27), for example, found a significant discordance between patients' preferences and end-of-life and life-sustaining intensive care in the very old. They also reported a high rate of prolonged ICU stay in old patients.

It is interesting that, despite the fact that the very old patients received less intensive care treatment than the old patients, even after adjustment for severity of illness, the mortality is only marginally higher. If could be that the more aggressive treatment in the old (the younger patients) may not have been indicated, and therefore it was not associated with an improved outcome. Alternatively, the very old patients may have received less intensive care and been discharged from the ICU for further palliative treatment–making them formally ICU survivors as their mortality would not have been captured in our analysis. If we had looked at longer term follow up, mortality may have been much higher in this patient group. In support of this, Biston et al. (28) showed that most very old (in this case, >85 years) patients had died one year after an admission with circulatory shock.

Due to the absolute mortality difference of 2% points in both groups, the number needed to “harm” would be 50. Therefore, based on these data, for patients with a chronological age over 80 years, we would not consider a generic withholding of intensive medical therapy to be justified. However, there may be other reasons or scenarios when withholding and “rationing” intensive care treatment may be deemed appropriate, especially for old people (29). Health economic considerations, which weigh the considerable costs of an intensive care stay with an associated high mortality on the one hand and the high morbidity among the survivors on the other, are understandable and necessary (30, 31). However, it is well established, especially in the health economics' literature, that it is not the chronological age, but the remaining life expectancy, that predicts cost and morbidity and that these two are not necessarily the same thing (32). Unfortunately, Mark Twain's *bonmot* that it is difficult to make predictions, especially about the future, applies to predicting the life expectancy of an individual patient.

This study aimed to look at mortality as the primary outcome. However, for older people who are close to their natural end of life, functional outcomes, such as quality of life are of equal or greater importance (33, 34). It is unclear to what extent modern intensive care medicine affects this (35, 36). The older (>64 years) and very old (>79 years) often have a high mortality in the event of an acute critical illness, and the functional outcomes of survivors are unclear (13–15). Nevertheless, survival represents an essential–and easy to measure–variable for outcome. Other important parameters, such as the need for care or frailty after intensive care, are not captured in eICU. Also, no data on quality-of-life are available in the database. Studies looking at long-term variables that reflect the quality-of-life of old patients after intensive care treatment are ongoing (37) (NCT04321265).

Intensive care medicine requires robust and reliable parameters which enable us to predict outcomes in intensive care, particularly when treatment is deemed futile. Established scores, such as SOFA, biomarkers, such as lactate and novel developments including machine learning algorithms are helpful (38–40). However, we think that these tools can only augment clinical judgement. One tool which nicely integrates clinical judgement, formal semi-quantification of functional capacity and a patient's risk of an adverse outcome is the evaluation of frailty (13, 14, 41–43). Several studies have shown that frailty is associated with an increased mortality. As frailty is more frequently present in the very old, it could contribute to the observed robust association of chronological age and mortality. Unfortunately, and this is a major limitation, we do not have data on the patients' frailty or functional capacity in this study.

Of note, in some subgroups we found a pronounced association between being very old and ICU mortality. In very old patients, mortality was higher in female patients and in those who did not receive mechanical ventilation or vasopressors. We are aware of the limitations of subgroup analyses (44), and we demonstrated recently that there were no clinically relevant differences between the sexes in septic patients (45, 46). However, the trend toward a higher mortality in patients that did not receive intubation or vasopressors could reflect a more restrictive use of this therapy in very old patients. This could be secondary to a justified limitation of therapy or an under use of these therapies in very old patients. Ultimately, these considerations remain speculative, as we have no data on whether treatment was withheld in these patients, which is another limitation of this study.

Although we found a higher mortality in the subgroup of patients with septic shock and numerically a higher mortality in the very old patients, even in this subgroup, the absolute difference between old and very old patients was 2%points, a level that we would not consider clinically relevant. In this subgroup, the functional outcomes would be of particular interest as after prolonged intubation and immobilization very old patients may suffer from significant morbidity resulting in the inability to lead an independent life. Unfortunately, we do not have data on this, another relevant limitation of our data.

Interestingly, and in contrast to previous studies, such as Heyland et al. (27), in our analysis, the length of ICU stay was shorter, and the rate of a long (>7 days) ICU stay was less in the very old patients. The analysis by Heyland et al. was performed in Canada, whereas the present data are from the United States, and local factors may have contributed to these differences. We do not know whether the shorter ICU stay in this cohort occurred because of “time limited ICU trials,” where initial intensive care treatment was provided for a defined period of time and due to lack of benefit the focus of care was changed to palliation, or due to other factors (47). Based on our data, the statement that intensive care in very old patients is primarily a “quality finish” seems incorrect and pessimistic (27).

It is open to debate to what extent this effect of chronological age on mortality, although statistically significant and detectable even after multi-variable correction, is clinically relevant. The absolute differences were, in our opinion, relatively small both in the total cohort of septic patients (13 vs. 11%), and in the subgroup of patients with septic shock (38 vs. 36%). From the clinicians' point of view, we interpret our data along with other preliminary studies in very old intensive care patients that the blanket denial of intensive care treatment based on the calendar age alone does not seem justifiable. From our perspective, a combination of pre-admission risk factors (such as frailty), markers of disease severity on admission (such as acute organ failure) and solid clinical judgement should be used to assess patients in all age categories in order to formulate a bespoke and realistic clinical plan. In the (frequent) case of ambiguity, an “ICU trial” is an opportunity to gain further information or at least time to consider the individual patient's prognosis (47, 48). We would like to appeal to our fellow clinicians not to make an association between chronological age and a worse outcome a “self-fulfilling prophecy,” and not to withhold therapies from very old patients based on chronologic age alone.

From a scientific perspective, the association of chronological age and intensive care outcomes seems less robust than perhaps intuitively assumed. However, due to the retrospective nature of this study these data do not allow for any generalization of the findings. With this analysis, we hope to gain a better understanding and to propose suggestions for future prospective studies evaluating this issue. Future studies should focus on different aspects of aging, such as frailty, and on different outcome measures, such as independent daily living or quality of life.

## Conclusion

This study found a 2% absolute difference in mortality between old and very old septic patients, which translates into a relative risk difference of ~20% in a vulnerable patient population. This finding is statistically significant but probably clinically irrelevant. This study underlines the pivotal importance of concepts, such as frailty that involve the biological age of patients and not the chronological age alone for outcome prediction. Based on these data, being old or very old alone are insufficient to define therapeutic goals.

## Data Availability Statement

The raw data supporting the conclusions of this article will be made available by the authors, without undue reservation.

## Ethics Statement

The study was an analysis of two third-party anonymized publicly available databases with a pre-existing institutional review board (IRB) approval. The database was released under the Health Insurance Portability and Accountability Act (HIPAA) safe harbor provision.

## Author Contributions

RB, BW, CJ, and BM analyzed the data and wrote the first drought of the manuscript. SL, HF, and VO contributed to statistical analysis and improved the paper. RR, SB, PB, GW, MK, BG, DDL, DD, AK, TD, WS, SS, PvH, and MB gave guidance and improved the paper. All authors read and approved the final manuscript.

## Conflict of Interest

The authors declare that the research was conducted in the absence of any commercial or financial relationships that could be construed as a potential conflict of interest.

## References

[1] Vincent JL Marshall JC Namendys-Silva SA Francois B Martin-Loeches I Lipman J. Assessment of the worldwide burden of critical illness: the intensive care over nations (ICON) audit. Lancet Respir Med. (2014) 2:380–6. 10.1016/S2213-2600(14)70061-X 24740011

[2] van Vught LA Klein Klouwenberg PM Spitoni C Scicluna BP Wiewel MA Horn J. Incidence, risk factors, and attributable mortality of secondary infections in the intensive care unit after admission for sepsis. JAMA. (2016) 315:1469–79. 10.1001/jama.2016.2691 26975785

[3] Sakr Y Moreira CL Rhodes A Ferguson ND Kleinpell R Pickkers P. The impact of hospital and ICU organizational factors on outcome in critically ill patients: results from the extended prevalence of infection in intensive care study. Crit Care Med. (2015) 43:519–26. 10.1097/CCM.0000000000000754 25479111

[4] Prescott HC Angus DC. Enhancing recovery from sepsis: a review. JAMA. (2018) 319:62–75. 10.1001/jama.2017.17687 29297082 PMC5839473

[5] Singer M Deutschman CS Seymour CW Shankar-Hari M Annane D Bauer M. The third international consensus definitions for sepsis and septic shock (Sepsis-3). JAMA. (2016) 315:801–10. 10.1001/jama.2016.0287 26903338 PMC4968574

[6] de Lange DW Brinkman S Flaatten H Boumendil A Morandi A Andersen FH. Cumulative prognostic score predicting mortality in patients older than 80 years admitted to the ICU. J Am Geriatr Soc. (2019) 67:1263–7. 10.1111/jgs.15888 30977911 PMC6850576

[7] Martin GS Mannino DM Moss M. The effect of age on the development and outcome of adult sepsis. Crit Care Med. (2006) 34:15–21. 10.1097/01.CCM.0000194535.82812.BA 16374151

[8] Flaatten H de Lange DW Artigas A Bin D Moreno R Christensen S. The status of intensive care medicine research and a future agenda for very old patients in the ICU. Intensive Care Med. (2017) 43:1319–28. 10.1007/s00134-017-4718-z 28238055

[9] Leblanc G Boumendil A Guidet B. Ten things to know about critically ill elderly patients. Intensive Care Med. (2017) 43:217–9. 10.1007/s00134-016-4477-2 27492269

[10] Ihra GC Lehberger J Hochrieser H Bauer P Schmutz R Metnitz B. Development of demographics and outcome of very old critically ill patients admitted to intensive care units. Intensive Care Med. (2012) 38:620–6. 10.1007/s00134-012-2474-7 22354500

[11] Beil M Sviri S Flaatten H De Lange DW Jung C Szczeklik W. On predictions in critical care: the individual prognostication fallacy in elderly patients. J Crit Care. (2021) 61:34–8. 10.1016/j.jcrc.2020.10.006 33075607 PMC7553132

[12] van Heerden PV Sviri S Beil M Szczeklik W de Lange D Jung C. The wave of very old people in the intensive care unit-A challenge in decision-making. J Crit Care. (2020) 60:290–3. 10.1016/j.jcrc.2020.08.030 32949896

[13] Flaatten H De Lange DW Morandi A Andersen FH Artigas A Bertolini G. The impact of frailty on ICU and 30-day mortality and the level of care in very elderly patients (>/= 80 years). Intensive Care Med. (2017) 43:1820–8. 10.1007/s00134-017-4940-8 28936626

[14] Guidet B de Lange DW Boumendil A Leaver S Watson X Boulanger C. The contribution of frailty, cognition, activity of daily life and comorbidities on outcome in acutely admitted patients over 80 years in European ICUs: the VIP2 study. Intensive Care Med. (2020) 46:57–69. 10.1007/s00134-019-05853-1 31784798 PMC7223711

[15] Jung C Wernly B Muessig JM Kelm M Boumendil A Morandi A. A comparison of very old patients admitted to intensive care unit after acute versus elective surgery or intervention. J Crit Care. (2019) 52:141–8. 10.1016/j.jcrc.2019.04.020 31055187

[16] Riva L Petrini C. Ethics of triage for intensive-care interventions during the COVID-19 pandemic: age or disability related cut-off policies are not justifiable. Clin Ethics. (2020). 10.1177/1477750920971803

[17] Pollard TJ Johnson AEW Raffa JD Celi LA Mark RG Badawi O. The eICU collaborative research database, a freely available multi-center database for critical care research. Sci Data. (2018) 5:180178. 10.1038/sdata.2018.178 30204154 PMC6132188

[18] Angus DC Linde-Zwirble WT Lidicker J Clermont G Carcillo J Pinsky MR. Epidemiology of severe sepsis in the United States: analysis of incidence, outcome, and associated costs of care. Crit Care Med. (2001) 29:1303–10. 10.1097/00003246-200107000-00002 11445675

[19] Marik PE Weinmann M. Optimizing fluid therapy in shock. Curr Opin Crit Care. (2019) 25:246–51. 10.1097/MCC.0000000000000604 31022087

[20] Taeb AM Hooper MH Marik PE. Sepsis: current definition, pathophysiology, diagnosis, and management. Nutr Clin Pract. (2017) 32:296–308. 10.1177/0884533617695243 28537517

[21] Ibarz M Boumendil A Haas LEM Irazabal M Flaatten H de Lange DW. Sepsis at ICU admission does not decrease 30-day survival in very old patients: a *post-hoc* analysis of the VIP1 multinational cohort study. Ann Intensive Care. (2020) 10:56. 10.1186/s13613-020-00672-w 32406016 PMC7221097

[22] Guidet B Aegerter P Gauzit R Meshaka P Dreyfuss D CUB-Réa Study Group. Incidence and impact of organ dysfunctions associated with sepsis. Chest. (2005) 127:942–51. 10.1378/chest.127.3.942 15764780

[23] Nasa P Juneja D Singh O Dang R Arora V. Severe sepsis and its impact on outcome in elderly and very elderly patients admitted in intensive care unit. J Intensive Care Med. (2012) 27:179–83. 10.1177/0885066610397116 21436163

[24] Martin-Loeches I Guia MC Vallecoccia MS Suarez D Ibarz M Irazabal M. Risk factors for mortality in elderly and very elderly critically ill patients with sepsis: a prospective, observational, multicenter cohort study. Ann Intensive Care. (2019) 9:26. 10.1186/s13613-019-0495-x 30715638 PMC6362175

[25] Boumendil A Aegerter P Guidet B CUB-Rea Network. Treatment intensity and outcome of patients aged 80 and older in intensive care units: a multicenter matched-cohort study. J Am Geriatr Soc. (2005) 53:88–93. 10.1111/j.1532-5415.2005.53016.x 15667382

[26] Guidet B Flaatten H Boumendil A Morandi A Andersen FH Artigas A. Withholding or withdrawing of life-sustaining therapy in older adults (>/= 80 years) admitted to the intensive care unit. Intensive Care Med. (2018) 44:1027–38. 10.1007/s00134-018-5196-7 29774388

[27] Heyland D Cook D Bagshaw SM Garland A Stelfox HT Mehta S. The very elderly admitted to ICU: a quality finish? Crit Care Med. (2015) 43:1352–60. 10.1097/CCM.0000000000001024 25901550

[28] Biston P Aldecoa C Devriendt J Madl C Chochrad D Vincent JL. Outcome of elderly patients with circulatory failure. Intensive Care Med. (2014) 40:50–6. 10.1007/s00134-013-3121-7 24132383

[29] Sprung CL Artigas A Kesecioglu J Pezzi A Wiis J Pirracchio R. The Eldicus prospective, observational study of triage decision making in European intensive care units. Part II: intensive care benefit for the elderly. Crit Care Med. (2012) 40:132–8. 10.1097/CCM.0b013e318232d6b0 22001580

[30] Breyer F Lorenz N. The “red herring” after 20 years: ageing and health care expenditures. Eur J Health Econ. (2020) 22:661–7. 10.1007/s10198-020-01203-x 32500244 PMC8214577

[31] Costa-Font J Vilaplana-Prieto C. 'More than one red herring'? Heterogeneous effects of ageing on health care utilisation. Health Econ. (2020) 29:8–29. 10.1002/hec.4035 32677116

[32] Zweifel P Felder S Meiers M. Ageing of population and health care expenditure: a red herring? Health Econ. (1999) 8:485–96. 10.1002/(sici)1099-1050(199909)8:6<485::aid-hec461>3.0.co;2-4 10544314

[33] Garrouste-Orgeas M Tabah A Vesin A Philippart F Kpodji A Bruel C. The ETHICA study (part II): simulation study of determinants and variability of ICU physician decisions in patients aged 80 or over. Intensive Care Med. (2013) 39:1574–83. 10.1007/s00134-013-2977-x 23765237

[34] Philippart F Vesin A Bruel C Kpodji A Durand-Gasselin B Garcon P. The ETHICA study (part I): elderly's thoughts about intensive care unit admission for life-sustaining treatments. Intensive Care Med. (2013) 39:1565–73. 10.1007/s00134-013-2976-y 23765236

[35] Hofmann JC Wenger NS Davis RB Teno J Connors AF Jr Desbiens N. Patient preferences for communication with physicians about end-of-life decisions SUPPORT investigators study to understand prognoses and preference for outcomes and risks of treatment. Ann Intern Med. (1997) 127:1–12. 10.7326/0003-4819-127-1-199707010-00001 9214246

[36] Heyland DK Barwich D Pichora D Dodek P Lamontagne F You JJ. Failure to engage hospitalized elderly patients and their families in advance care planning. JAMA Intern Med. (2013) 173:778–87. 10.1001/jamainternmed.2013.180 23545563

[37] Jung C Flaatten H Fjolner J Bruno RR Wernly B Artigas A. The impact of frailty on survival in elderly intensive care patients with COVID-19: the COVIP study. Crit Care. (2021) 25:149. 10.1186/s13054-021-03551-3 33874987 PMC8054503

[38] Wernly B Mamandipoor B Baldia P Jung C Osmani V. Machine learning predicts mortality in septic patients using only routinely available ABG variables: a multi-centre evaluation. Int J Med Inform. (2021) 145:104312. 10.1016/j.ijmedinf.2020.104312 33126059

[39] Wernly B Heramvand N Masyuk M Rezar R Bruno RR Kelm M. Acidosis predicts mortality independently from hyperlactatemia in patients with sepsis. Eur J Intern Med. (2020) 76:76–81. 10.1016/j.ejim.2020.02.027 32143899

[40] Wernly B Lichtenauer M Franz M Kabisch B Muessig J Masyuk M. Model for end-stage liver disease excluding INR (MELD-XI) score in critically ill patients: easily available and of prognostic relevance. PLoS ONE. (2017) 12:e0170987. 10.1371/journal.pone.0170987 28151948 PMC5289507

[41] Flaatten H Beil M Guidet B. Prognostication in older ICU patients: mission impossible? Br J Anaesth. (2020) 125:655–7. 10.1016/j.bja.2020.08.005 32868042 PMC7427523

[42] Flaatten H Beil M Guidet B. Elderly patients in the intensive care unit. Semin Respir Crit Care Med. (2020) 42:010–9. 10.1055/s-0040-1710571 32772353

[43] Abraham P Courvoisier DS Annweiler C Lenoir C Millien T Dalmaz F. Validation of the clinical frailty score (CFS) in French language. BMC Geriatr. (2019) 19:322. 10.1186/s12877-019-1315-8 31752699 PMC6873717

[44] Horton R. From star signs to trial guidelines. Lancet. (2000) 355:1033–4. 10.1016/S0140-6736(00)02031-6 10744086

[45] Wernly B Bruno RR Mamandipoor B Jung C Osmani V. Sex-specific outcomes and management in critically ill septic patients. Eur J Intern Med. (2021) 83:74–7. 10.1016/j.ejim.2020.10.009 33059966

[46] Wernly B Bruno RR Kelm M Boumendil A Morandi A Andersen FH. Sex-specific outcome disparities in very old patients admitted to intensive care medicine: a propensity matched analysis. Sci Rep. (2020) 10:18671. 10.1038/s41598-020-74910-3 33122713 PMC7596065

[47] Vink EE Azoulay E Caplan A Kompanje EJO Bakker J. Time-limited trial of intensive care treatment: an overview of current literature. Intensive Care Med. (2018) 44:1369–77. 10.1007/s00134-018-5339-x 30136140

[48] Guidet B Leblanc G Simon T Woimant M Quenot JP Ganansia O. Effect of systematic intensive care unit triage on long-term mortality among critically ill elderly patients in France: a randomized clinical trial. JAMA. (2017) 318:1450–9. 10.1001/jama.2017.13889 28973065 PMC5710364

